# Exploring heart rate variability as a biomarker for frailty: A narrative review

**DOI:** 10.1111/eci.70194

**Published:** 2026-03-27

**Authors:** Luca Tagliafico, Silvia Ottaviani, Riccardo Balzano, Stefania Peruzzo, Alessio Nencioni, Fiammetta Monacelli

**Affiliations:** ^1^ Section of Geriatrics, Department of Internal Medicine and Medical Specialties (DiMI) University of Genoa Genoa Italy; ^2^ IRCCS Ospedale Policlinico San Martino Genoa Italy

**Keywords:** aging biomarkers, autonomic nervous system, digital health, frailty assessment, HRV, physiological reserve

## Abstract

**Background:**

Frailty is defined as a state of increased vulnerability to stressors resulting from reduced physiological homeostasis. The need to identify biomarkers capable of offering insight into the biological underpinnings of frailty and supporting early detection, diagnosis, and prognostic evaluation has grown in recent years. Heart rate variability (HRV), defined as the temporal variability between consecutive R waves of the electrocardiogram (R‐R intervals), has emerged as a promising physiological biomarker in this context.

**Methods:**

We conducted a narrative review of studies to synthesize evidence on HRV and frailty, examining analytical domains of HRV and situating findings within the current biomarker framework. Relevant literature was assessed across time‐domain, frequency‐domain, geometric and non‐linear measures of HRV, together with reported associations involving clinical, functional and cognitive outcomes.

**Results:**

Within the landscape of frailty biomarkers, HRV provides a non‐invasive index of autonomic nervous system function. Reduced HRV has been associated with frailty status, diminished physiological reserve, adverse clinical outcomes, and cognitive decline, with particularly notable findings when assessed during physiological challenges rather than at rest. It should be noted, however, that the literature is heterogeneous and methodologically inconsistent, especially regarding recording duration and the specific HRV parameters analysed.

**Conclusions:**

HRV is a highly promising physiological biomarker in the context of frailty, but further research is needed to clarify several aspects of its role. Its potential integration with other biomarkers may help confirm its validity and could contribute to identifying biomarker clusters relevant to personalized approaches.

## Graphical abstract

Frailty arises from progressive multisystem dysregulation, prompting interest in biomarkers that capture declining physiological reserve. This review positions heart rate variability (HRV) within this evolving biomarker landscape, outlining its physiological basis, analytical domains and relevance to clinical research. Evidence indicates that reduced HRV reflects impaired autonomic–cardiovascular regulation and is associated with frailty, cognitive impairment and adverse health outcomes. HRV thus emerges as an accessible, integrative marker to support multimodal frailty assessment. Created in https://BioRender.com.

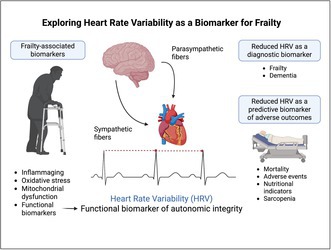

## 1 INTRODUCTION

In recent decades, advances in medical and technological knowledge, as well as in public health, have led to an increase in life expectancy among the populations worldwide.^1^ However, the aging of the population is often associated with several issues, like an increase in the prevalence of chronic diseases, physical disabilities and cognitive disorders.^2^

The aging process and the onset of the aforementioned issues vary greatly between individuals, reflecting considerable heterogeneity, spanning from what is considered successful aging, including the absence of physically disabling conditions and chronic diseases, to advanced clinical scenarios often described as pathological aging.^3^ In this view, chronological age is a poor indicator of the complexity of these processes.

It is, therefore, crucial to develop assessment methods that enable subjects' stratification while offering a repeatable and easily communicable evaluation of health status in the aging population. Such methods can significantly enhance care appropriateness and support the identification of personalized care plans for each individual, according to their will also.

Frailty, in this regard, represents a fundamental aspect. It is defined as a state of increased vulnerability to stressors, resulting from reduced physiological homeostasis, and, therefore, associated with negative outcomes.^4^, ^5^ In fact, frail people are at higher risk of acute events, hospitalization, loss of independence, falls, worsening of comorbidities and mortality.^4^, ^6^ It can also be regarded as a clinical manifestation of an individual's biological age, offering more meaningful insights than chronological age.^7^

As a geriatric syndrome, its nature is inherently multifactorial, encompassing a wide range of aspects, from psychosocial dimensions to specific diseases, patterns of multimorbidity, and biological processes closely linked to the mechanisms of aging.^8^, ^9^, ^10^

The main methods for assessing frailty derive from models described in the literature.

A first approach is the model proposed by Linda P. Fried and colleagues, which defines the so‐called frailty clinical phenotype, characterized by a specific set of signs and symptoms. In particular, the criteria include unintended weight loss, exhaustion, weakness, slowness and low physical activity.^11^

According to this model, a patient is classified as frail when three or more of these criteria are present, whereas the presence of one or two criteria indicates a pre‐frail or vulnerable condition.^11^

Another relevant model of frailty is based on the deficit accumulation approach of Kenneth Rockwood and colleagues. This model considers a range of health deficits potentially associated with aging, whose accumulation is linked to an increased risk of adverse outcomes.^12^ In practice, a series of evaluations, ranging from the original 70 items to shorter versions, is performed and the total number of identified deficits is divided by the total number of items assessed.^12^ Moreover, additional scales have been developed from this model, most notably the Clinical Frailty Scale, which stratifies patients from ‘very fit’ to ‘terminally ill’ through a shorter and more practical assessment method.^13^

These models and their respective assessment methods are not necessarily contradictory; rather, they can be potentially complementary and useful in different contexts.^14^ Additionally, each assessment method has specific limitations; for instance, the frailty index requires specialist expertise and involves time‐consuming tests,^15^ while the frailty phenotype risks oversimplifying the complex underlying biology of frailty to only its most common signs and symptoms.^16^

From this perspective, there arises a clear need to evaluate biomarkers of frailty, especially those linked to its pathophysiology. They are particularly relevant for several reasons: they can enhance our understanding of the complexity of frailty, including its implications and potential targets for interventions, thereby supporting more detailed pre‐clinical research.^17^ They may also be predictive biomarkers, allowing for the early and simple identification of susceptible patients, or diagnostic biomarkers, allowing for frailty identification by themselves or included in a broader clinical characterization.^18^ Finally, biomarkers can play a valuable role in assessing patient prognosis in the context of frailty, helping to estimate the risk of its progression or, more broadly, the likelihood of adverse outcomes.^19^

Biomarkers associated with aging and with frailty can be of several classes, including molecular, digital and functional or physiological.^20^

One of the most studied categories is definitely molecular biomarkers, based mainly on hallmarks of aging, including those associated with processes like age‐related low‐grade chronic inflammation (‘inflammaging’), mitochondrial dysfunction, cellular senescence, oxidative stress and modifications of DNA methylation.^21^, ^22^

Moreover, digital biomarkers are studied, and they are becoming increasingly relevant in the current literature through the use of sensors, wearable devices, and other digital technologies.^23^ These tools offer the advantage of being non‐invasive and scalable for use in large patient populations, providing reasonable diagnostic accuracy that could enable the early identification of frailty and related conditions.^23^

Other relevant biomarkers in the field of frailty are physiological or functional ones. These biomarkers can be measures of functional performance or physical characteristics of the patient.^20^ Heart Rate Variability (HRV), which refers to the variation over time in the intervals between successive heartbeats, measured as the consecutive R waves of the electrocardiogram (R‐R intervals), is a relevant physiological biomarker and is going to be the focus of the literature review.^24^, ^25^

The aim of this narrative review is to summarize the main findings of the literature regarding the role of HRV in frailty and associated geriatric syndromes, particularly dementia. We will evaluate the identified literature encompassing the evidence of HRV in this clinical context and the issues and limitations of the current literature, giving, in conclusion, potential future directions of research in this regard.

## 2 METHODS

A structured literature search was conducted in PubMed/MEDLINE and Scopus to identify original studies investigating HRV as a predictor, biomarker or prognostic indicator across frailty, cognitive, and broader clinical outcomes. Searches covered the period from 1st January 2010 to the 9th February 2026 and were restricted to human studies. In PubMed, HRV was searched in the title field and combined with terms related to frailty, cognitive impairment, dementia, clinical outcomes and age‐related conditions. In Scopus, HRV was searched in the title field and combined with relevant keywords in titles, abstracts and indexed terms. A structured literature search was conducted in PubMed/MEDLINE and Scopus to identify original studies investigating HRV as a predictor, biomarker or prognostic indicator across frailty, cognitive and clinical outcomes. PubMed/MEDLINE was searched using the following string:

(‘heart rate variability’[Title] AND (frailty OR ‘frailty index’ OR dementia OR ‘cognitive impairment’ OR Alzheimer* OR mortality OR prognosis OR hospitalization OR ‘heart failure’ OR stroke OR delirium OR sarcopenia OR ‘physical performance‘)) NOT (review[Publication Type]) NOT (animals[MeSH Terms] NOT humans[MeSH Terms]).

The Scopus search included TITLE (‘heart rate variability’) combined with TITLE‐ABS‐KEY terms related to frailty, cognitive impairment, dementia, mortality, prognosis, hospitalization, sarcopenia or older adults.

**FIGURE 1:**
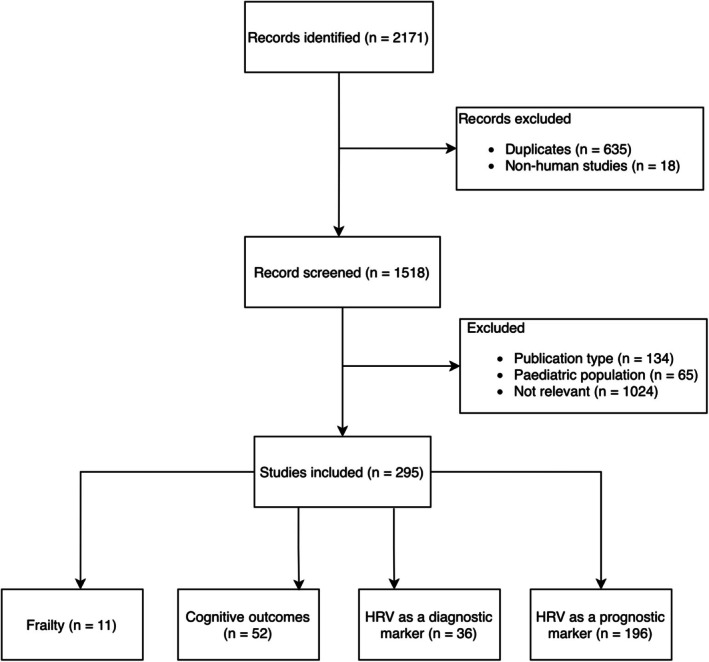
Literature search flowchart and thematic distribution of included HRV studies.

As depicted in Figure 1, a total of 2,171 records were identified (PubMed/MEDLINE *n* = 872; Scopus *n* = 1,299). After automatic deduplication, 1547 records remained. During title and abstract screening, 11 additional duplicates and 18 veterinary studies were removed. Studies were excluded if they were of inappropriate publication type (*n* = 134), focused on paediatric populations (*n* = 65), or did not investigate HRV as a predictor or biomarker (e.g., HRV used solely as an outcome measure; *n* = 1024).

The remaining studies were grouped thematically into: HRV and frailty (*n* = 11); HRV and cognitive outcomes, including delirium (*n* = 52); HRV as a diagnostic biomarker across diseases (*n* = 36); and HRV as a prognostic or severity biomarker (*n* = 188).

Given the large number and heterogeneity of eligible studies, detailed tabulation was restricted to those specifically investigating frailty, which represented the primary focus of the present work (see Table 1).

**TABLE 1:** Overview of studies investigating HRV in relation to frailty.

	Study (year)	Country	Design and population	Inclusion criteria	Exclusion criteria	Frailty assessment	HRV assessment	HRV indices	Main findings	Confounders considered	Limitations
1	Alvarez‐Millan (2022)^26^	Mexico	Cross‐sectional comparative study; young (19–29 years), middle‐aged (30–59 years), frail (>60 years) and non‐frail (>60 years) adults (total *n* = 80)	Ability to walk 160 m independently	Atrial fibrillation; medications affecting autonomic function; inability to perform walking test; discordant frailty classification	FRAIL scale and CFS	Chest‐strap ECG (Bioharness 3.0); 5‐min standing rest (baseline) and 5‐min recovery after 160‐m walking test	SDNN, RMSSD, pNN30, pNN50, LF power (nu), HF power (nu), LF/HF, SD1, SD2, SampEn, ApEn, DFA‐α1	Baseline (frail vs. non‐frail): ↑ HF power (nu), ↓ LF power (nu), ↓ LF/HF. Recovery: ↓ SampEn, altered DFA‐α1, delayed heart‐rate recovery. ↔ frequency‐domain indices between baseline and recovery.	Age group stratification; no multivariable adjustment for comorbidities	Cross‐sectional design; small sample size; short‐term HRV; no multivariable adjustment
2	Chen (2023)^27^	Hong Kong	Cross‐sectional study; community‐dwelling adults ≥50 years (non‐frail *n* = 33; prefrail *n* = 10)	Age ≥50 years; ambulatory status	Major cognitive impairment; structural heart disease; COPD, cancer or recent stroke; implanted pacemaker; tricyclic antidepressant use	Modified Physical Frailty Phenotype (mPFP)	Wearable chest‐belt HRV device; 5‐min recordings in standing, sitting and lying positions	SDNN, RMSSD, LF, HF, LF/HF	At rest (within postures): ↔ HRV indices between groups. Postural transitions: ↑ RMSSD, HF and LF/HF only in non‐frail individuals; ↔ modulation in prefrail participants.	Partial adjustment for age, gender and comorbidities (binary variable)	Small sample size; absence of frail participants; wearable‐derived HRV; convenience sampling
3	Dewangan (2024)^28^	India	Cross‐sectional observational study; older adults ≥60 years recruited from geriatric outpatient clinic (non‐frail *n* = 70; pre‐frail *n* = 70; frail *n* = 70)	Age ≥60 years	Critical illness; primary neurological disease affecting autonomic nervous system; cardiac pacemaker; arrhythmias	36‐item Frailty Index	5‐min resting ECG (standard limb leads) after 15‐min supine rest	SDNN, SDSD, rMSSD, NN50, pNN50, LF power (absolute), HF power (absolute), LF/HF, SD1, SD2, SD1/SD2	Frail vs. pre‐/non‐frail: ↓ SDNN, SDSD, RMSSD, NN50, pNN50, LF power, HF power, SD1, SD2. ↔ LF/HF, SD1/SD2. Frailty Index: negative correlations with SDNN, RMSSD, LF, HF, SD1 and SD2.	No formal multivariable adjustment	Cross‐sectional clinical sample; hospital‐based population; comorbidity imbalance
4	Kanamanapalli (2025)^29^	India	Cross‐sectional study; patients with Type 2 Diabetes Mellitus aged 50–65 years (*n* = 139; non‐frail = 7, prefrail = 55, frail = 77)	Type 2 diabetes mellitus; age 50–65 years	Endocrine, cardiovascular, renal, pulmonary, psychiatric disorders or malignancy	Frailty Phenotype	5‐min resting ECG (supine); short‐term HRV analysis according to Task Force standards	SDNN, RMSSD, NN50, pNN50, LF power (absolute), HF power (absolute), Total Power, LF power (nu), HF power (nu), LF/HF	Frail vs. pre‐/non‐frail: ↓ SDNN, RMSSD, NN50, pNN50, Total Power, HF power. ↑ LF power (nu), LF/HF. ↔ LF power (absolute).	Stratification by diabetes duration and physical activity; no multivariable modelling	Cross‐sectional tertiary‐care sample; small non‐frail subgroup; univariate analyses only
5	Li (2020)^30^	China	Retrospective observational study; elderly ≥65 years Orthostatic hypotension (OH) *n* = 108 vs. controls *n* = 64	Age ≥ 65 years; clinically stable	Arrhythmias, pacemaker, diabetes, drugs affecting autonomic function	Groningen Frailty Indicator, 30‐item Frailty Index	24‐h Holter ECG; Task Force–based HRV analysis (GE MARS software)	SDNN, SDANN, rMSSD, pNN50, LF, HF, VLF, ULF, LF/HF	OH vs. controls: ↓ SDNN, LF, VLF, LF/HF. ↔ SDANN, RMSSD, pNN50, HF. Frailty Index: higher in OH group and independently associated with OH.	Age, gender, smoking, drinking wine, BMI, resting blood pressure, resting heart rate, fast blood sugar, LDL hypertension, hyperlipidemia, hyperuricemia, tumours, and cerebral infarction	Retrospective design; disease‐specific population; single‐center cohort
6	Mabe‐Castro (2024)^31^	Chile	Cross‐sectional observational study; community‐dwelling older adults, *n* = 81	Age ≥ 60 years; community‐dwelling older adults	Congenital heart disease; beta‐blocker use; stimulant substances within 24 h; motor or cognitive disability; pain during testing	No formal frailty tool; physical performance assessed with SPPB and TMST	Chest‐strap HRV monitoring; 5‐min recordings at rest, during and after exercise	RMSSD, SDNN; composite autonomic indices (SNS index, PNS index, Stress Index)	During exercise: ↑ SNS index and heart rate, ↓ RMSSD and PNS index with higher physical fitness. ↑ RMSSD and SDNN, ↓ SNS index and Stress Index with higher body fat percentage.	Sex‐stratified analyses; mediation models including fitness, muscle mass and psychological variables; no adjustment for major clinical comorbidities.	Cross‐sectional design; small sample size; predominantly female sample; no validated frailty tool
7	Ogliari (2015)^32^	UK, Ireland, Netherlands	Prospective cohort study (PROSPER trial); older adults aged 70–82 years (*n* = 5042)	Age 70–82 years; total cholesterol 4.0–9.0 mmol/L; pre‐existing vascular disease or ≥1 major vascular risk factor (hypertension, smoking or diabetes).	Atrial fibrillation or major arrhythmias; pacemaker; severe cognitive impairment (MMSE <24); advanced heart failure; inability to attend visits	No formal frailty tool; functional status used as a proxy (ADL and IADL)	Resting 10‐s 12‐lead ECG	SDNN	Lower SDNN: ↓ IADL performance at baseline. Longitudinally: ↓ SDNN predicted higher risk of ADL/IADL decline over 3.2 years.	Age, sex, education, smoking, BMI, diabetes, hypertension, vascular disease history and multiple cardiovascular medications	Ultra‐short ECG duration; single HRV metric; high cardiovascular‐risk cohort
8	Samuel (2025)^33^	Canada	Cross‐sectional clinical study; ambulatory cardiovascular patients; *n* = 155; mean age 67 years; 44% female	Ambulatory cardiology patients with valid HRV recordings and frailty assessment	Arrhythmias, pacemaker rhythm or poor‐quality recordings leading to unreliable HRV signals	CFS; frailty defined as CFS ≥5	Finger‐based photoplethysmography; 2.5‐min seated resting recording	RMSSD, SDNN, lnRMSSD, pNN50, mean R‐R, LF power, HF power, LF/HF ratio, HF peak, LF peak, Total Power	Frail vs. non‐frail: ↓ LF/HF ratio, ↓ LF power, ↑ HF peak. Multivariable analysis: ↓ LF/HF independently associated with higher CFS scores.	Age, sex, blood pressure, heart rate, cardiovascular comorbidities, beta‐blocker use	Ultra‐short recording (2.5 min); PPG‐derived HRV; cross‐sectional cardiovascular clinic sample
9	Soares‐Miranda (2014)^34^	US	Prospective longitudinal cohort study (Cardiovascular Health Study); community‐dwelling adults ≥65 years (*n* = 985)	Age ≥65 years; ambulatory status; community‐dwelling adults	Institutionalization; markedly irregular cardiac rhythms; insufficient usable Holter data; missing physical activity data	No formal frailty assessment; physical activity measures (leisure‐time activity, walking distance and pace)	24‐h Holter ECG (baseline and 5‐year follow‐up)	SDNN, SDNN index, RMSSD; ULF, VLF, NLF, NHF, LF/HF; DFA1, Poincaré ratio (SD12)	Higher physical activity: ↑ SDNN, ↑ ULF. Faster walking pace: ↑ DFA1, ↓ Poincaré ratio (SD12). ↔ rMSSD, NLF, NHF, LF/HF.	Age, sex, race, education, income, smoking, alcohol intake, diet, BMI, blood pressure, cardiovascular disease, diabetes and cardiovascular medications	Observational cohort; self‐reported physical activity; no validated frailty definition
10	Turcu (2025)^35^	Romania	Observational cross‐sectional study; hospitalized older adults ≥65 years; *n* = 83; mean age 75.6 years; 63.9% female	Age ≥65 years; intrinsic capacity deficit or pre‐frailty/frailty	Age <65 years; lack of informed consent; inability to obtain clinical or biochemical data; bedridden status or inability to stand; presence of pacemaker	Frailty phenotype; ADL, IADL; intrinsic capacity domains (nutrition, locomotion, cognition, psychological)	24‐h Holter ECG	SDNN, SDANN	Higher frailty/poorer ADL: ↓ SDANN. Intrinsic capacity domains (nutrition, locomotion, psychological): associations with SDNN.	No formal multivariable adjustment (univariable analyses only)	Small single‐center hospitalized cohort; no multivariable adjustment; cross‐sectional analyses
11	Yu (2022)^36^	Germany	Pilot observational study on frail geriatric inpatients undergoing early geriatric rehabilitation (*n* = 8 analysed) and healthy community‐dwelling older adults (*n* = 9)	Age >70 years; frail inpatients undergoing early geriatric rehabilitation and healthy community‐dwelling older adults.	Pacemaker; atrial fibrillation; insufficient mobility to participate in physiotherapy	Frailty phenotype	Non‐contact PPGI; short‐term 5‐min resting HRV with ECG reference validation	LF/HF ratio	Frail vs. non‐frail: ↑ LF/HF ratio at baseline. Post‐rehabilitation: ↓ LF/HF ratio. PPGI‐derived HRV trends consistent with ECG reference at group level.	No multivariable adjustement	Very small pilot sample; single HRV index; short‐term recordings; limited confounder control

Abbreviations: ADL, activities of daily living; ApEn, approximate entropy; CFS, clinical frailty scale; COPD, chronic obstructive pulmonary disease; DFA‐α1, detrended fluctuation analysis alpha‐1; ECG, electrocardiogram; HF, high frequency power; HRV, heart rate variability; IADL, instrumental activities of daily living; LF, low frequency power; LF/HF, low‐to‐high frequency ratio; MNA, mini nutritional assessment; mPFP, modified physical frailty phenotype; NN50, number of successive NN intervals differing by more than 50 ms; nu, normalized units; pNN50, percentage of successive NN intervals differing by more than 50 ms; PNS, parasympathetic nervous system index; PPGI, photoplethysmography imaging; RMSSD, root mean square of successive differences; SD1/SD2, poincaré plot indices; SDANN, standard deviation of the average NN intervals; SDNN, standard deviation of NN intervals; SNS, sympathetic nervous system index; SPPB, short physical performance battery; TMST, two‐minute step test; ULF, ultra low frequency power; VLF, very low frequency power.

The remaining literature predominantly investigated HRV as a prognostic or disease severity marker (*n* = 196), spanning a wide range of clinical contexts, most commonly stroke or haemorrhage (*n* = 27), cancer (*n* = 25), chronic kidney disease or haemodialysis (*n* = 19), diabetes (*n* = 18), adverse events in hospitalized populations (*n* = 18), myocardial infarction (*n* = 16), sepsis (*n* = 13), including heart failure or atrial fibrillation (*n* = 11), with smaller groups addressing cirrhosis, COVID‐19, surgery, traumatic brain injury, rheumatologic conditions and Parkinson's disease.

Studies focusing on cognitive outcomes accounted for 52 articles, primarily examining cognitive performance (*n* = 18), risk of mild cognitive impairment or dementia (*n* = 15), and delirium (*n* = 11), whereas fewer explored neuroimaging or biomarker correlates (*n* = 5), behavioural and psychological symptoms of dementia (*n* = 2), or subjective cognitive decline (*n* = 1).

A smaller body of literature evaluated HRV as a diagnostic biomarker (*n* = 36), most frequently in stress, anxiety, or depression (*n* = 9) and cardiovascular conditions (*n* = 5), followed by sarcopenia (*n* = 4), stroke or haemorrhage (*n* = 3), Parkinson's disease (*n* = 3), diabetes (*n* = 2), sepsis (*n* = 2), fatigue or fibromyalgia (*n* = 2) and falls (*n* = 2).

## 3 RESULTS

### 3.1 Heart rate variability (HRV)

HRV, as introduced above, represents the temporal variability between successive R‐R intervals,^24^, ^25^ and reflects the natural ability of the brain to modulate oscillations in heart rate. HRV thus provides a non‐invasive window into the brain–heart axis and the individual's capacity to adapt to internal and external stressors.

As we will illustrate in the following paragraphs, based on the selected literature, HRV can be conceptualized as a functional biomarker of autonomic integrity, as a diagnostic or monitoring biomarker to detect early autonomic impairment associated with frailty and dementia, and as a prognostic biomarker for adverse outcomes related to frailty, age‐related chronic diseases and other geriatric syndromes.

### 3.2 Pathophysiology of HRV

HRV is an important parameter for assessing the functionality of the autonomic nervous system (ANS)^37^ and is a proxy of the effectiveness of interactions between the heart, central nervous system (CNS) and ANS, while also being continuously influenced by respiration, baroreflex sensitivity and arterial blood pressure.^24^, ^25^

At the cardiac level, the ANS operates through a network of intrinsic ganglionic cells that integrate both sympathetic and parasympathetic inputs, as well as interneuronal and efferent circuits connecting to sympathetic and parasympathetic neurons. Such an intrinsic cardiac nervous system also processes sensory information from internal organs, blood vessels, and cardiomyocytes, allowing dynamic regulation of heart rate according to current physiological demands.^38^

Sympathetic fibres (originating from the T1–T4 vertebral segments) synapse in the cervical and thoracic ganglia and innervate the atria, ventricles, and cardiac nodes. Noradrenaline, by binding to β‐adrenergic receptors, increases the concentration and effectiveness of intracellular Ca^2+^, leading to accelerated depolarization in the sinoatrial node, faster conduction in the atrioventricular node and greater myocardial contractile force. This results in positive inotropic and chronotropic effects.

**FIGURE 2:**
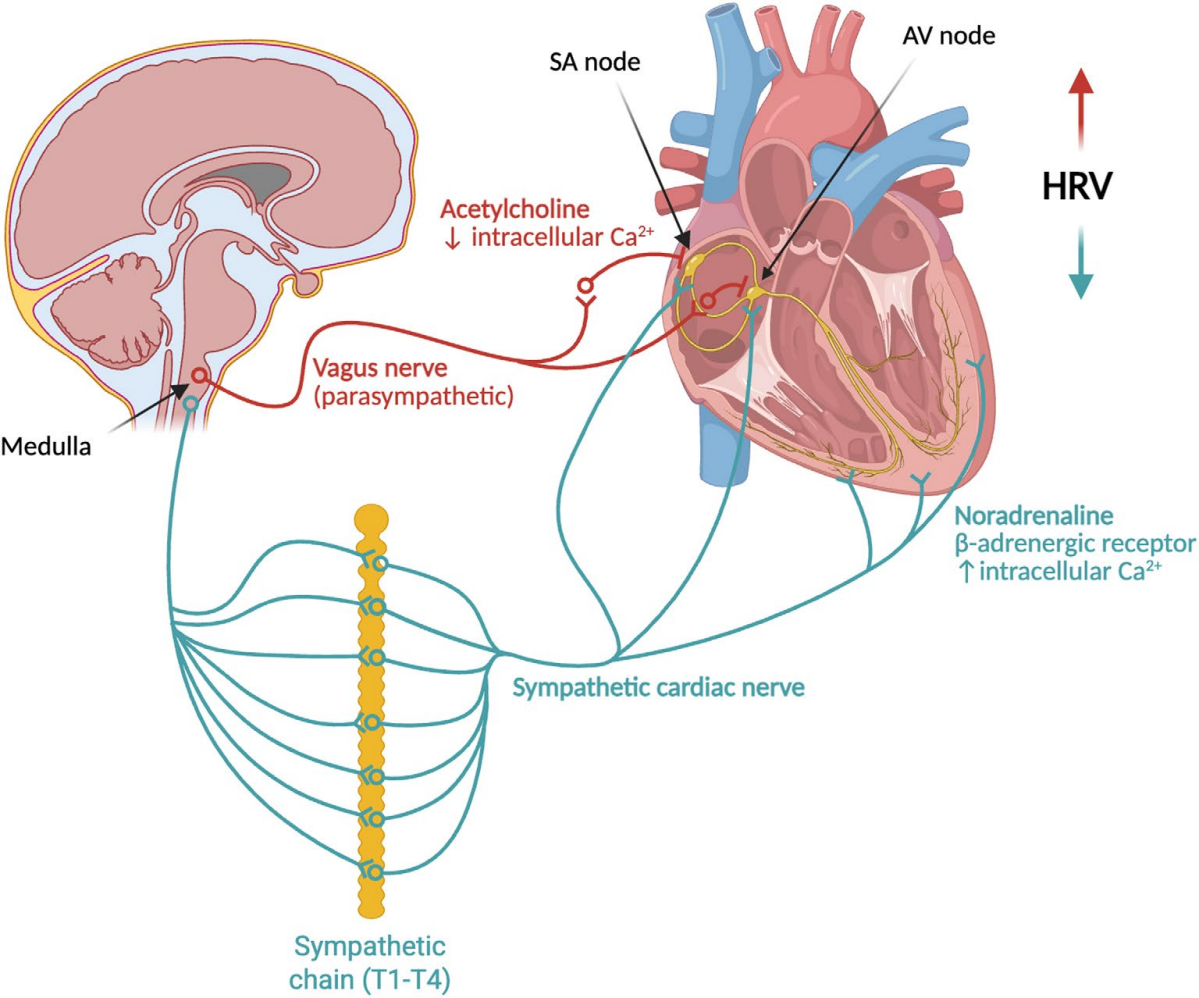
Autonomic control of heart rate variability. Schematic representation of the central autonomic regulation of HRV. Sympathetic cardiac fibres originating from the thoracic sympathetic chain (T1–T4) release noradrenaline acting on β‐adrenergic receptors, increasing intracellular Ca^2+^ and promoting positive chronotropic and inotropic effects. In contrast, parasympathetic vagal efferents arising from the medulla release acetylcholine at the sinoatrial node, reducing intracellular Ca^2+^ and slowing heart rate. The dynamic interaction between sympathetic and parasympathetic inputs at the sinoatrial (SA) and atrioventricular (AV) nodes generates beat‐to‐beat fluctuations in heart rate, reflected as HRV. AV, atrioventricular; Ca^2+^, calcium ion; HRV, heart rate variability; SA, sinoatrial; T1–T4, thoracic spinal segments 1–4. Created in https://BioRender.com.

Parasympathetic fibres (originating from the vagus nerve) synapse in the cardiac plexus; the right postganglionic fibres innervate the right atrium and the sinoatrial node, while those on the left innervate the left atrium, the atrioventricular node and the bundle of His. Parasympathetic ventricular innervation is sparse. Acetylcholine, by inhibiting adenylate cyclase, reduces intracellular Ca^2+^ and produces effects opposite to those of the sympathetic nervous system. The inotropic effect is minimal due to limited ventricular innervation. Continuous vagal activity determines parasympathetic tone: high tone induces bradycardia, while reduced tone promotes tachycardia. Intense stimulation can slow the sinoatrial node to the point of activating ectopic foci^25^, ^38^, ^39^ (see Figure 2).

During aging, deterioration of ANS function can impair the brain's capacity to regulate cerebral blood flow effectively.^40^ This reduction in neurovascular control, compounded by age‐related structural vascular changes, has been associated with cognitive decline and neurodegenerative or cerebrovascular conditions such as Alzheimer's disease (AD)^41^ and stroke.^42^ The same autonomic dysfunction also affects cardiac regulation, leading to reduced complexity and variability of heart rate dynamics. Consequently, decreased HRV may reflect an impaired capacity to respond to physiological and environmental stressors.

Beyond autonomic imbalance, evidence suggests that HRV may also relate to aerobic fitness and long‐term cardiovascular resilience: exercise interventions improve vagal‐mediated HRV indices,^43^ and parasympathetic HRV measures have been associated with aerobic capacity in some populations,^44^ although findings remain inconsistent in healthy subjects.^45^ From a lifespan perspective, reduced HRV and higher heart rate have been linked to increased mortality and aging‐related cardiovascular risk,^46^, ^47^ while HRV indices such as SDNN have shown prognostic value for survival in very old‐age individuals and centenarians.^48^, ^49^

In this perspective, the decline in ANS efficiency at the cardiac level underlies a loss of complexity, implying that HRV analysis might be a valuable non‐invasive diagnostic marker for estimating frailty, as well as a prognostic marker for predicting hospitalization, mortality, and cardiovascular complications in older adults.^50^

In addition to reduced physiological complexity, chronic low‐grade inflammation may represent a key biological pathway linking autonomic imbalance to frailty. As previously discussed, chronic low‐grade inflammation is strongly associated with frailty, but recent evidence suggests that such a process is also tightly interconnected with autonomic nervous system regulation. Existing literature indicates that inflammatory activation interacts closely with autonomic regulation: higher inflammatory biomarkers have been associated with increased heart rate and reduced HRV even in healthy populations,^51^ while global reductions in HRV have been observed during pro‐inflammatory states and infection.^52^ Reduced vagal activity may impair neuroimmune modulation through the cholinergic anti‐inflammatory pathway, reinforcing a bidirectional loop between autonomic dysfunction and systemic inflammation.^53^ Clinical observations further support this interaction, as lower HRV has been linked to greater inflammatory burden and functional impairment in chronic inflammatory conditions,^54^ and inflammatory markers have been associated with frailty status in type 2 diabetes.^55^ Within this framework, HRV may reflect the interplay of autonomic dysregulation and inflammatory burden underlying frailty trajectories.

### 3.3 HRV evaluation

To standardize HRV evaluation, several analytical domains have been established (cf. Table 2):

**TABLE 2:** Analytical domains and metrics of HRV.

Domain	Metric	Interpretation
Time‐domain	SDNN SDANN	Overall autonomic modulation
	RMSSD SDSD	Short‐term, vagally mediated variability
	NN50/pNN50	Beat‐to‐beat variability and parasympathetic modulation
Frequency‐domain	HF (0.15–0.40 Hz)	Parasympathetic modulation
	LF (0.04–0.15 Hz)	Debated, for mixed sympathetic–parasympathetic influences
	VLF (<0.04 Hz)	Slower regulatory mechanisms (e.g., RAAS, peripheral vasomotor tone)
	LF/HF ratio	Sympatho‐vagal balance index
Non‐linear methods	Poincaré SD1, SD2	Short‐term (SD1) and long‐term (SD2) autonomic variability
	SampEn, ApEn	Complexity and irregularity of R‐R interval dynamics.
	DFA	Fractal scaling and long‐range correlations

Abbreviations: ApEn, approximate entropy; DFA, detrended fluctuation analysis; HF, high‐frequency power; LF, low‐frequency power; NN50, number of RR interval pairs differing by >50 ms; pNN50, percentage of R‐R interval pairs differing by >50 ms; RAAS, renin–angiotensin–aldosterone system; RMSSD, root mean square of successive differences; SampEn, sample entropy; SD, standard deviation; SDANN, standard deviation of mean R‐R intervals; SDNN, standard deviation of normal‐to‐normal R‐R intervals; SDSD, standard deviation of successive differences; VLF, very‐low‐frequency power.

*Time‐domain parameters* are based on R‐R interval analysis. The most commonly used are the standard deviation of all R‐R intervals (SDNN) and the standard deviation of the averages of R‐R intervals calculated over short periods (SDANN, typically 5‐minute segments). Both reflect combined sympathetic and parasympathetic modulation, although short‐term recordings are more influenced by parasympathetic activity. Other indices include the root mean square of successive differences between adjacent R‐R intervals (RMSSD), which primarily reflects vagal activity; the standard deviation of these successive differences (SDSD); the number of interval differences greater than 50 ms (NN50), and its percentage relative to the total number of intervals (pNN50). SDNN and SDANN are mainly used in long‐term recordings, while NN50, pNN50 and SDSD have shown significant correlations with frailty even in short‐term analyses.^28^

*Frequency‐domain parameters* are derived from spectral analysis and quantify the power of high‐frequency (HF, 0.15–0.4 Hz), low‐frequency (LF, 0.04–0.15 Hz) and very‐low‐frequency (VLF, <0.04 Hz) components. HF power predominantly reflects parasympathetic activity, whereas LF has been traditionally attributed to sympathetic modulation. However, more recent evidence suggests that LF variability also contains a parasympathetic component, making this interpretation controversial.^25^, ^56^, ^57^, ^58^, ^59^ VLF power is thought to be related to renin–angiotensin–aldosterone system activity or peripheral vasomotor tone. The LF/HF ratio is often used to estimate sympatho‐vagal balance,^60^ although its reliability is debated due to the mixed origin of LF oscillations. Frequency‐domain parameters are generally more reliable in long‐term recordings, while their validity in short or ultra‐short segments remains limited.^25^, ^57^

*Other analytical approaches* include geometric and non‐linear methods. Geometric indices evaluate the distribution density of R‐R intervals, whereas non‐linear methods, such as the Poincaré plot and its derived standard deviations (SD1 and SD2), assess parasympathetic activity. Measures of data complexity and regularity, such as sample entropy (SampEn) and approximate entropy (ApEn), provide additional insights into vagal modulation,^25^, ^61^ while detrended fluctuation analysis (DFA) evaluates how heart rate patterns change over time.

Recent studies have extended HRV analysis to dynamic conditions such as exercise; in a study of late middle‐aged adults undergoing 14 days of head‐down bed rest, two DFA‐derived HRV indices (HRV_0.75_ and HRV_0.5_) closely tracked changes in ventilatory thresholds and oxygen uptake, showing sensitivity to both deconditioning and exercise.^62^ These findings suggest that HRV may serve as an accessible, non‐invasive marker of cardiorespiratory fitness.

### 3.4 Methodological challenges in HRV assessment

Despite the growing use of HRV, methodological heterogeneity remains a major limitation for clinical interpretation. Recording duration represents a key source of variability, as short‐term and long‐term measurements reflect different physiological processes and cannot be used interchangeably.^56^, ^63^ Normative ranges for common HRV indices also show wide dispersion across studies, partly driven by differences in preprocessing and spectral analysis methods.^64^ Signal acquisition further contributes to inconsistency: while ECG remains the reference standard, wearable‐derived HRV based on photoplethysmography may introduce bias related to motion artefacts and proprietary algorithms.^65^ In addition, variability in analytic pipelines and incomplete reporting of acquisition conditions remain common challenges, reinforcing the need for harmonized protocols and consensus methodological standards.^66^ These methodological issues also affect the interpretability of HRV metrics in clinical settings. Indices most frequently reported, such as SDNN, RMSSD and HF power, are influenced by recording duration, analytical approach, and population characteristics, which may limit direct comparison across studies. In addition, widely accepted normative cut‐offs are lacking, and reference values often depend on specific methodological conditions. Therefore, HRV parameters should be interpreted cautiously and within the context of the acquisition protocol and study population.

### 3.5 Overview of the HRV clinical implications

There is extensive evidence highlighting the crucial role of ANS and its interaction with the heart in the onset and risk stratification of unfavourable health outcomes, such as cerebrovascular events, arrhythmias, acute heart failure and transient ischemic attacks.

Several studies have demonstrated a significant correlation between reduced HRV and increased mortality, particularly in patients with chronic cardiovascular disease^67^, ^68^ or recent myocardial infarction.^69^ Similar associations have been reported in non‐cardiac conditions, including liver cirrhosis, independent of MELD score,^68^ renal failure and cancer,^70^ as well as among older adults hospitalized for cardiovascular surgery^71^ or respiratory infections.^72^, ^73^ Reduced HRV has also been linked to higher mortality in patients on dialysis,^74^ in old‐age individuals with type II diabetes,^75^ and even in the general population, with alterations observed in both time‐ and frequency‐domain parameters.^70^, ^76^

Beyond mortality, numerous studies have reported associations between reduced HRV and various pathological conditions and risk factors, highlighting its possible role as a diagnostic biomarker. These include decompensated type 2 diabetes mellitus,^77^ in which some authors describe autonomic dysfunction preceding the onset of the disease without a clear causal link,^78^ while others identify diabetic neuropathy as a contributing factor to HRV reduction and as a predictor of secondary complications such as retinopathy and pressure ulcers.^79^, ^80^ In geriatric populations, regular physical activity and good glycemic control are positively correlated with HRV,^80^, ^81^ supporting its potential role as an informative biomarker of healthy aging and physiological resilience.

Malnutrition has been associated with decreased HRV and lower nutritional indicators,^82^, ^83^ whereas adherence to a balanced diet correlates with greater HRV.^84^ Likewise, obesity,^82^, ^85^ sarcopenia, reduced muscle strength, and poor physical performance have been consistently linked to lower HRV parameters, as demonstrated by functional tests (e.g., hand‐grip strength), imaging findings and biochemical markers.^86^, ^87^, ^88^, ^89^

Additional studies have reported associations between reduced HRV and postural instability,^90^ acute cerebrovascular events,^91^ in‐hospital delirium^71^ and psycho‐affective disorders such as depression, anxiety and low self‐esteem.^92^, ^93^ Conversely, higher HRV appears to serve as an informative biomarker of physiological resilience, reflecting more effective regulation of cardiac and inflammatory responses to external stressors.^25^

**FIGURE 3:**
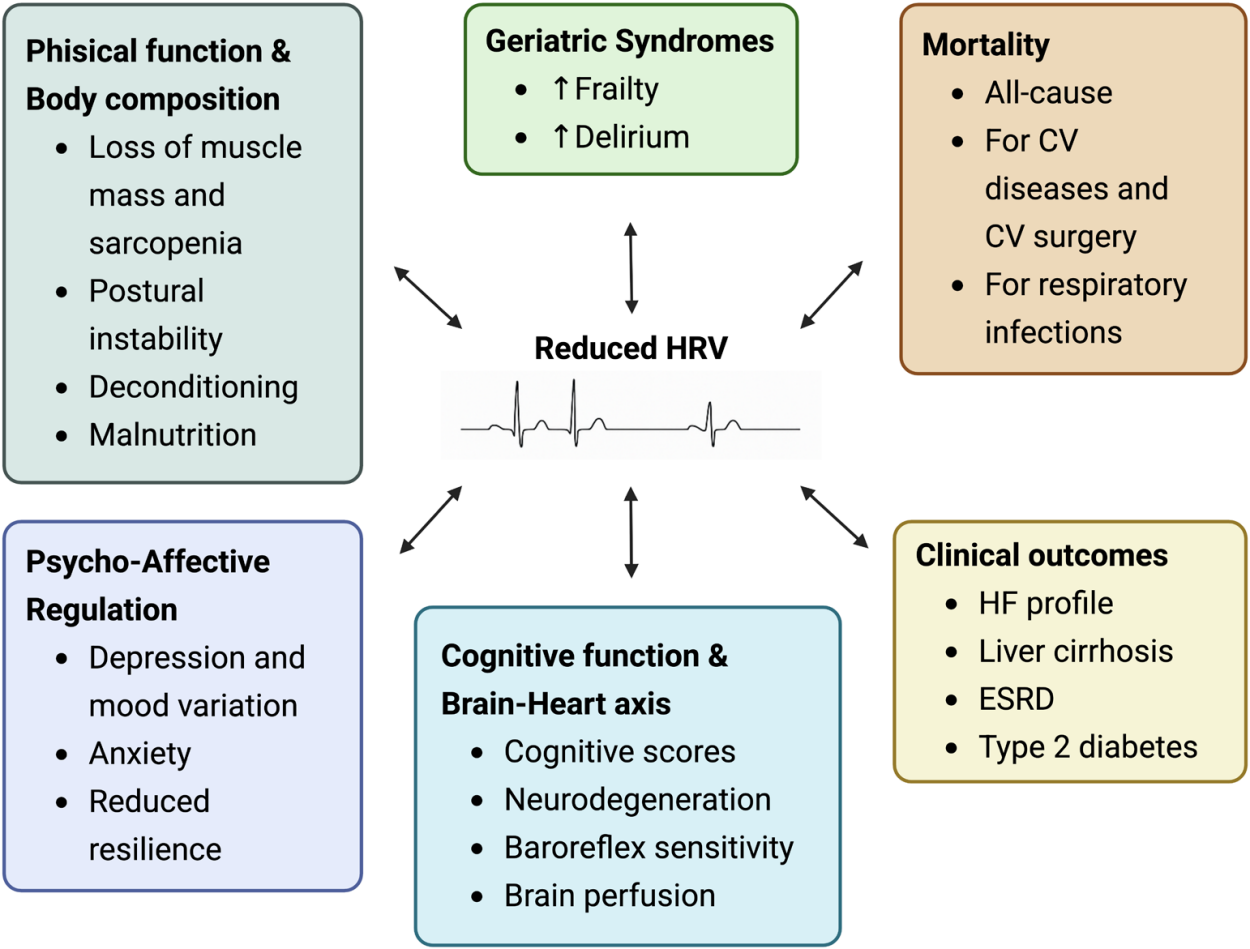
Clinical Implications of Reduced HRV. Schematic overview of the main clinical domains in which reduced HRV has been associated with adverse outcomes. Evidence from the literature indicates that lower HRV reflects impaired autonomic regulation and is linked to alterations in physical function and body composition (including sarcopenia and deconditioning), psycho‐affective dysregulation (depression, anxiety, reduced resilience), cognitive dysfunction and brain–heart axis alterations, geriatric syndromes such as frailty and delirium, as well as increased morbidity and mortality across cardiovascular, metabolic, infectious and systemic conditions. The diagram summarizes the breadth of clinical contexts identified in the literature and does not imply direct causality. Domains are not mutually exclusive and frequently overlap in clinical practice. CV, cardiovascular; ESRD, end‐stage renal disease; HF, heart failure; HRV, heart rate variability. Created in https://BioRender.com.

For an overall view of HRV's clinical implications, see Figure 3.

### 3.6 HRV as a biomarker for frailty

The ANS, particularly its cardiac efferent branch, plays a pivotal role in maintaining homeostasis under stress. As previously discussed, this ability is impaired in frail individuals. It is therefore unsurprising that autonomic dysfunction has been identified as altered across the frailty spectrum.

Early evidence from community‐based cohorts showed that frailty is associated with lower HRV and reduced complexity of cardiac rhythms, suggesting the concept of impaired autonomic regulation underlying clinical vulnerability.^94^ Experimental findings further reinforced this biological plausibility: in a murine model, Dorey and colleagues demonstrated that reduced HRV across multiple domains was linked to higher frailty index scores and altered sympatho‐vagal balance.^95^

Across clinical studies summarized in Table 1, HRV has primarily been explored as a cross‐sectional marker of frailty status rather than as a diagnostic tool. Several works report lower time‐domain HRV indices across increasing frailty levels,^28^, ^29^ whereas findings for frequency‐domain measures, particularly LF/HF ratio, are inconsistent across populations and recording protocols. Notably, some studies indicate that autonomic differences become more apparent during physiological challenges rather than resting recordings, such as postural transitions,^27^ or recovery after physical stress.^26^ These discrepancies likely reflect differences in frailty definitions, acquisition methods, recording duration, and disease‐specific cohorts.

Evidence supporting a prognostic role of HRV in frailty is even more limited. In a large prospective cohort, lower short‐term SDNN predicted subsequent decline in functional status,^32^ while observational data linking HRV with physical activity and functional performance suggest potential longitudinal relevance but often rely on proxy measures rather than validated frailty instruments.^34^ In line with this heterogeneity, a recent three‐level meta‐analysis by Chen and colleagues found a consistent trend toward lower HRV values in pre‐frail compared to non‐frail older adults, particularly in time‐domain parameters, which showed greater sensitivity than frequency‐domain indices; however, they highlighted substantial methodological discrepancy and non‐significant pooled effects, underscoring the uncertainty about HRV as a standalone biomarker of frailty.^96^ Attenuation of differences after stratification by frailty stage further suggests that autonomic alterations may emerge early along the frailty trajectory but are difficult to disentangle from clinical overlap between pre‐frailty and frailty.

Overall, current evidence suggests that HRV captures aspects of autonomic dysregulation associated with frailty, but its diagnostic and prognostic utility remains context‐dependent. Variability in frailty assessment, HRV acquisition, and confounder adjustment across studies limits comparability and likely contributes to divergent findings, supporting a careful interpretation of HRV within the broader clinical framework.

### 3.7 HRV as a biomarker for dementia

The close interplay between frailty and dementia further supports this perspective.^97^ Both conditions share common pathophysiological mechanisms—such as chronic inflammation, vascular dysfunction, and impaired autonomic regulation—that contribute to reduced physiological reserve. Longitudinal studies have shown that frailty trajectories often accelerate years before the onset of dementia, suggesting that frailty may act as both a precursor and an amplifier of neurodegenerative processes.^98^ Conversely, cognitive decline can exacerbate physical vulnerability, reinforcing a bidirectional relationship between the two syndromes.

In the field of dementia research, evidence consistently points to autonomic dysfunction, particularly altered HRV and cerebral blood flow regulation, not only in vascular dementia (VaD) but also in AD, dementia with Lewy bodies (LBD), and Parkinson's disease‐dementia (PD‐D).^99^ This autonomic imbalance typically manifests as sympathetic hyperactivity coupled with reduced parasympathetic tone.^99^, ^100^

Systematic reviews and meta‐analyses have confirmed the presence of autonomic dysfunction across the major forms of neurocognitive disorders. Lin and colleagues demonstrated that resting high‐frequency HRV (HF‐HRV) and HF‐HRV reactivity are significantly correlated with greater neurodegeneration on structural imaging, reduced activity of the anterior cingulate cortex on functional imaging and poorer cognitive performance as assessed by the Montreal Cognitive Assessment.^101^ De Pablo‐Fernandez and colleagues, in a retrospective study of 100 patients with pathologically confirmed PD, showed that early autonomic dysfunction, clinically identified through urinary, gastrointestinal, orthostatic and sudomotor symptoms, was associated with faster disease progression and shorter survival, suggesting autonomic assessment as a potential prognostic marker.^102^ Similarly, Nicolini and colleagues, in a cross‐sectional study of 292 individuals, reported significant alterations in autonomic function (assessed through HRV frequency‐domain analysis and additional non‐HRV parameters) among patients with both amnestic (aMCI) and non‐amnestic (naMCI) mild cognitive impairment. These alterations correlated with lower cognitive scores, reduced functional autonomy and both structural and functional brain damage, particularly in the naMCI group, further supporting the role of autonomic dysfunction as an early prognostic and risk marker.^103^

From a pathophysiological standpoint, two main hypotheses have been proposed to explain the relationship between autonomic dysfunction and dementia: acetylcholine deficiency and altered cerebral perfusion. Neurotransmitter deficits are consistently observed across major neurocognitive disorders, and together with advanced brain atrophy, they may further impair autonomic flexibility. The locus coeruleus–noradrenaline system has also been implicated as a potential link between abnormal tau pathology and sympathetic hyperactivity.^104^

On the other hand, the brain's high metabolic demand requires constant oxygen and nutrient supply, which is tightly regulated by the ANS through cerebral autoregulation and neurovascular coupling.^103^ While gradual blood pressure changes have minimal effect, rapid fluctuations can influence cerebral perfusion. Sympathetic and parasympathetic innervation of cerebral vessels contributes to protective regulation in response to acute hemodynamic changes.^99^ Recent neuroimaging studies have clarified how autonomic dysfunction may contribute to cognitive decline through impaired regulation of cerebral blood flow. Song and colleagues demonstrated that aging weakens the relationship between HRV, respiration and brain activity measured by the blood oxygenation level–dependent (BOLD) signal, particularly in regions that control autonomic and vascular functions such as the insula and anterior cingulate cortex. Interestingly, HRV biofeedback training partially restored these brain–body coupling patterns, suggesting that altered HRV–BOLD dynamics may reflect early neurovascular dysregulation involved in the pathogenesis of dementia.^105^

In AD, baroreceptor sensitivity declines progressively from cognitively healthy states to early cognitive impairment and further in overt AD. Treatment with acetylcholinesterase inhibitors (AChEIs) has been shown to improve baroreceptor function by approximately 66%, suggesting a mechanism through enhanced autonomic regulation.^106^ However, it remains unclear whether autonomic dysfunction is a cause or consequence of AD.^99^

### 3.8 Confounding factors in HRV research

Interpretation of HRV as a biomarker requires careful consideration of factors that independently influence autonomic regulation. Cardiovascular disease represents a major confounder, as reduced HRV is well documented across coronary disease, heart failure, and hypertension and baseline HRV varies according to cardiovascular risk burden even in population‐based cohorts.^107^, ^108^ Metabolic conditions, particularly diabetes and higher body mass index, further contribute to autonomic dysfunction and may partially account for the lower HRV observed in frail populations.^107^, ^109^ Psychiatric comorbidity, including depression and anxiety, has been consistently associated with reduced vagally mediated HRV indices, complicating interpretation in studies where affective symptoms overlap with frailty and cognitive impairment.^110^, ^111^ Polypharmacy represents an additional source of bias, as increasing medication burden has been linked to lower global HRV measures independent of disease status.^112^

Biological variability also plays a substantial role. Sex‐related differences in HRV have been reported across multiple cohorts, while ethnic diversity contributes to variation in normative HRV ranges.^107^, ^108^, ^113^ Moreover, HRV follows a pronounced circadian rhythm, and alterations in diurnal autonomic patterns have been described in pre‐frailty, highlighting the importance of recording protocols and timing when comparing studies.^114^, ^115^ Together, these factors emphasize that HRV is a sensitive but non‐specific marker of autonomic function, and inadequate adjustment for comorbidities, medications and biological variability may contribute to the heterogeneity observed across studies.

## 4 DISCUSSION

In this manuscript, we examined the potential role of HRV as a marker of ANS integrity with a focus on frailty and another strongly associated geriatric syndrome, dementia. From the literature search, HRV emerges as a core dimension of physiological resilience, given its ability to recapitulate the dynamic adaptation of the brain‐heart axis to stressors.

This is well exemplified by the data showing reduced HRV consistently correlates with increased mortality, hospitalization and adverse outcomes. Moreover, higher HRV has been associated with better physical performance,^86^, ^87^, ^88^, ^89^ greater aerobic capacity,^44^ healthier metabolic profiles,^80^, ^81^ and improved psychological well‐being.^92^, ^93^

However, despite consistent biological plausibility, its clinical translation remains limited patients by methodological heterogeneity and contextual dependency.

This is evident in the context of frailty, where a strong biological rationale gives the reduced capacity of frail patients to overcome stressors, but the clinical data in the literature are highly heterogeneous.

Evidence linking HRV and frailty is biologically coherent but methodologically heterogeneous. Most studies demonstrate lower time‐domain HRV indices across increasing frailty levels, whereas frequency‐domain findings remain inconsistent, particularly regarding the LF/HF ratio. Notably, autonomic differences often emerge more clearly during physiological challenges, such as postural transitions^27^ or stress recovery,^26^ suggesting that dynamic testing may be more informative than resting measurements.

However, current data predominantly derive from cross‐sectional designs, limiting causal inference. Longitudinal evidence remains scarce, and there are methodological discrepancies in the literature. Thus, while HRV appears to reflect autonomic dysregulation associated with frailty, it cannot yet be considered a standalone diagnostic biomarker. Instead, it may be better conceptualized as a complementary physiological indicator within multidimensional frailty assessment frameworks.

More robust data are available in the literature regarding the relationship between HRV and dementia, with also a connection with the pathophysiology of several neurodegenerative diseases regarding impaired cerebral autoregulation, neurotransmitter deficits and disrupted brain‐body coupling.

## 5 FUTURE DIRECTIONS

HRV is a highly promising physiological biomarker, although further research is needed to clarify several aspects of its role.

Longitudinal studies to clarify temporal relationships between HRV decline, frailty progression and cognitive impairment together with standardized acquisition protocols, including dynamic and stress‐based paradigms that may better capture autonomic reserve, would greatly improve the current knowledge.

Another important area for future research concerns the role of HRV within specific multimorbidity patterns, given its broad involvement across several diseases, as described in the Results section. It is plausible that HRV may be particularly relevant in specific clusters of comorbidities. In addition, the potential influence of polypharmacy and other confounding factors should be further explored to better define the comprehensive role of HRV in frail patients.

Another area of interest, where evidence is still limited, concerns the integration of HRV with a broader spectrum of frailty biomarkers, in particular, other physiological and molecular ones. This could be relevant for at least two reasons. First, it could help confirm the validity of HRV as a biomarker, either on its own or in combination with others, for the purposes described above. Second, it may allow the identification of biomarker clusters in specific patient subgroups, which could support more personalized approaches and contribute to uncovering the pathophysiological mechanisms underlying frailty.

Finally, integrating HRV with a more comprehensive series of digital biomarkers, given the increasing feasibility of electrocardiogram monitoring through telemedicine, could enhance the early identification of frail patients, provided that this approach is appropriately validated.

## 6 CONCLUSIONS

HRV, which reflects the dynamic interaction between circulatory, neurovascular and inflammatory control, is a physiologically reasonable and therapeutically useful indicator of autonomic integrity. Reduced HRV seems to indicate decreased adaptive reserve and loss of physiological complexity in the setting of aging, frailty, and dementia.

However, rather than serving as a conclusive diagnostic or prognostic tool, the evidence currently available supports its potential usage mainly as a supplementary biomarker, as part of a thorough geriatric assessment. HRV has the potential to become an important part of precision geriatric medicine by connecting multidimensional aging trajectories with cardiovascular physiology through methodological harmonization and long‐term validation.

## AUTHOR CONTRIBUTIONS

*Conceptualization*: Riccardo Balzano, Fiammetta Monacelli. *Literature review*: Riccardo Balzano, Luca Tagliafico, Silvia Ottaviani. *Writing—original draft preparation*: Luca Tagliafico, Silvia Ottaviani. *Writing—review and editing*: Stefania Peruzzo, Alessio Nencioni, Fiammetta Monacelli. *Supervision*: Alessio Nencioni, Fiammetta Monacelli. All authors have read and agreed to the published version of the manuscript.

## CONFLICT OF INTEREST STATEMENT

The authors declare no conflicts of interest.

## Data Availability

This article is a review and does not include any original data. All data referenced are from previously published sources, which are appropriately cited within the manuscript.
